# Apolipoprotein E is a pancreatic extracellular factor that maintains mature β-cell gene expression

**DOI:** 10.1371/journal.pone.0204595

**Published:** 2018-10-10

**Authors:** Ahmed I. Mahmoud, Francisco X. Galdos, Katherine A. Dinan, Mark P. Jedrychowski, Jeffrey C. Davis, Ana Vujic, Inbal Rachmin, Christian Shigley, James R. Pancoast, Samuel Lee, Jennifer Hollister-Lock, Catherine M. MacGillivray, Steven P. Gygi, Douglas A. Melton, Gordon C. Weir, Richard T. Lee

**Affiliations:** 1 Department of Stem Cell and Regenerative Biology and the Harvard Stem Cell Institute, Harvard University, Cambridge, MA, United States of America; 2 Department of Cell Biology, Harvard Medical School, Boston, MA, United States of America; 3 Islet Cell and Regenerative Biology Section, Joslin Diabetes Center, Harvard Stem Cell Institute, Harvard Medical School, Boston, MA, United States of America; Broad Institute, UNITED STATES

## Abstract

The *in vivo* microenvironment of tissues provides myriad unique signals to cells. Thus, following isolation, many cell types change in culture, often preserving some but not all of their *in vivo* characteristics in culture. At least some of the *in vivo* microenvironment may be mimicked by providing specific cues to cultured cells. Here, we show that after isolation and during maintenance in culture, adherent rat islets reduce expression of key β-cell transcription factors necessary for β-cell function and that soluble pancreatic decellularized matrix (DCM) can enhance β-cell gene expression. Following chromatographic fractionation of pancreatic DCM, we performed proteomics to identify soluble factors that can maintain β-cell stability and function. We identified Apolipoprotein E (ApoE) as an extracellular protein that significantly increased the expression of key β-cell genes. The ApoE effect on beta cells was mediated at least in part through the JAK/STAT signaling pathway. Together, these results reveal a role for ApoE as an extracellular factor that can maintain the mature β-cell gene expression profile.

## Introduction

The *in vivo* microenvironment provides necessary signals for maintenance of cell function and phenotype [1]. Cell-matrix and cell-cell interactions are often required for maintenance of a stable and mature cell phenotype [2–4]. The important role of the in vivo microenvironment may be demonstrated once cells are removed from their native environment. A notable example is the insulin-secreting β-cell, which has an extracellular matrix (ECM) environment that provides the cells with important biochemical signals and mechanical support that are required for β-cell survival and function [5–9]. Survival and function of adherent β-cells improve when cultured with extracellular matrix (ECM) proteins [10–13], demonstrating a role for extracellular signals. Furthermore, studies suggest that the loss of a stable β-cell phenotype leads to β-cell dedifferentiation either to a progenitor state or a different cell type, and this is a potential disease mechanism in the development of diabetes [14–17].

Advances in culture methods of pancreatic islets have improved their functionality *in vitro* [18]; however, the microenvironment signals that support islets *in vivo* are not yet completely understood. Interestingly, the capacity of cultured islets to re-establish normoglycemia in mice is significantly lower compared to fresh islets [5]. Furthermore, the glucose-stimulated insulin secretion (GSIS) response is also reduced in adherent cultures of pancreatic islets compared to fresh islets, suggesting that the culture conditions could be further enhanced by identifying important signals within the *in vivo* microenvironment [5, 6]. Previous studies suggested that adherent cultures of β-cells show improved functionality and viability when cultured with extracellular matrix components in both two-dimensional and three-dimensional cultures [19–23]. However, identifying local microenvironment signals that can maintain a mature β-cell state is challenging since the *in vivo* microenvironment is comprised of myriad biochemical and structural cues, not to mention the exocrine pancreatic cells that are quick to release degradative enzymes.

Here, we aim to identify the role of the soluble pancreatic extracellular proteins and determine their capacity to enhance the mature gene expression profile of β-cells. Furthermore, we aim to purify the soluble extracellular factor(s) that contribute to the enhanced mature β-cell state. These results will elucidate the role of the pancreatic microenvironment and identify the extracellular factors that can maintain a mature β-cell gene expression profile.

## Materials and methods

### Animals

All experiments were conducted in accordance with the Guide for the Use and Care of Laboratory Animals and approved by the Harvard Medical School Standing Committee on Animals. Sprague-Dawley (SD) rats were obtained from Charles River Laboratories. Rats were kept on a 12-h light/12-h dark cycle with water and food ad libitum. 100 rats were involved in this study. Rats were euthanized with carbon dioxide, followed by a secondary physical method of euthanasia.

### Plating of dissociated pancreatic islets in adhered culture

Rat pancreatic islets were centrifuged for 5 minutes at 1,500 rpm. Islets were washed with PBS and were again centrifuged for 5 minutes at 1,500 rpm. To dissociate the pancreatic islets, 1 mL of trypsin was added to the islets, which was pipetted for 2 minutes. Islets were kept at 37°C for 3 minutes to ensure complete dissociation of islet cells. Islets were then pipetted for 1 minute. Trypsin was neutralized with DMEM containing 2% fetal bovine serum. Cells were centrifuged for 5 minutes at 1,500 rpm, and resuspended in 10 mL of low glucose DMEM containing 2% fetal bovine serum. 200,000 islet cells were plated per well on 48-well plates.

### Pancreatic islet isolation

SD rat pancreatic islets were isolated as described previously [24]. Islet isolation was done by collagenase digestion of rat pancreata, which yielded ~10,000 islet equivalents per isolation. For assured purity of islets, islets were handpicked under a dissecting microscope and provided in 37 mL RPMI 1640 media with 10% fetal newborn calf serum.

### Plating of whole islets and treatments

Rat pancreatic islets were centrifuged for 5 minutes at 1,500 rpm. Islets were washed with 10 mL of 1x of sterile phosphate buffered saline (PBS) and were again centrifuged for 5 minutes at 1,500 rpm. For adherent cultures, islets were resuspended in 2% serum low glucose media, while islets cultured in suspension were resuspended in RPMI 1640 10% serum media. Approximately 100 islets were plated per well in 48 culture plates. β-cell culture media was replaced every 48 hours throughout course of time in culture. Cells were treated with either 1–2 μg/mL human recombinant ApoE (R&D systems), 20 uM JAK2 inhibitor (Tyrphostin AG490, Sigma), 2.5 uM STAT3 inhibitor (Pp-YLKTK-mts, Millipore), 20 ug LDL Receptor Blocking Peptide (Cayman Chemical), 50 mg of Cholesterol in 1 g of methyl β-cyclodextrin (Sigma), 22R-Hydroxycholesterol 10 uM (Sigma), Simvastatin 1uM (Sigma), 2 units/ml of Heparinase III (Sigma) and 500 uM Palmitate (Sigma).

### Decellularization of rat pancreata

Rat pancreas was decellularized as previously described [25]. For details, check S1 File in the supporting information.

### Gel filtration fractionation of digested DCM

Gel filtration chromatography was performed on solubilized decellularized matrix solutions in order to separate total proteins in digested DCM by protein size. For details, see S1 File in the supporting information.

### Glucose Stimulated Insulin Secretion (GSIS)

Whole islets were sampled for GSIS response. For adherent cultures, 100 islets equivalents were handpicked for each biological replicate assayed for the experiment. For details, check S1 File in the supporting information.

### Mass spectrometry identification of proteins

Prior to the submission of protein fractions for mass spectrometry, a methanol- chloroform precipitation of proteins in each fraction was done as previously described [26]. Trypsin digested DCM fractions were submitted for mass spectrometry. After precipitation, fractions were dried completely and stored at -80°C. Mass spectrometry identification of peptides was performed using triple-stage mass spectrometry (MS3) for the identification of peptides as described [27, 28].

### Viability staining

Islets were placed in a 6 cm dish with 10 mls of PBS, and Fluorescein Diacetate (FDA, Sigma) and Propidium Iodide (PI, Sigma) were added. Numbers of dead cells (PI positive) were divided by the total number of cells (1500 X number of islets (10–12 islets per group)).

### Phospho explorer antibody array

Protein samples from ApoE treated islets and controls were used for the Phospho Explorer Antibody Assay (Full Moon BioSystems, CA). This assay performed a quantitative protein phosphorylation profiling of over 30 signaling pathways for control and ApoE treated islets. A list of upregulated phosphorylated proteins was obtained.

### Statistical analysis

All data are presented as mean ± SEM. Student’s unpaired t test was used for comparisons between two groups unless otherwise noted. A value of P < 0.05 was considered significant.

## Results

### Downregulation of β-cell gene expression *in vitro*

**Fig 1:**
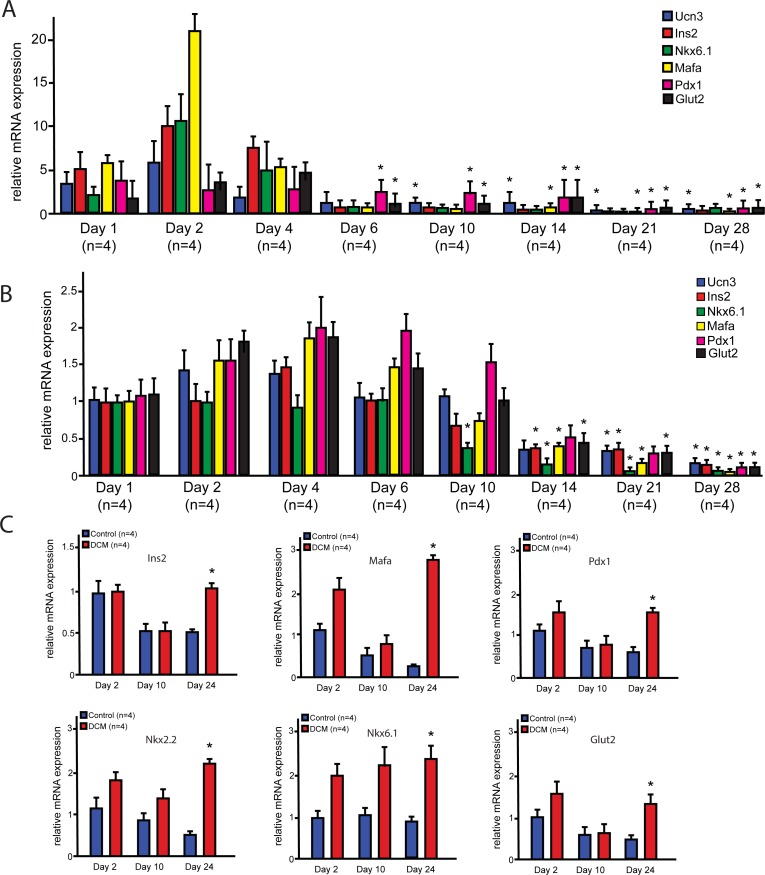
Pancreatic decellularized matrix (DCM) proteins maintains the expression of β-cell gene expression *in vitro*. **A)** Gene expression analysis of β-cell transcription factors from dissociated rat pancreatic islets cultured under low glucose (5 mM) conditions for 4 weeks (n = 4). **B)** Gene expression analysis of β-cell transcription factors from whole rat pancreatic islets cultured under physiological glucose (11 mM) conditions for 4 weeks (n = 4). **C)** Dissociated islet cells were plated on cell culture plates coated with whole solubilized pancreatic decellularized matrix under low glucose (5 mM) conditions. Gene expression was analyzed on days 2, 10, and 24 of culture relative to non- coated controls. Whole DCM increased the expression of key β-cell transcription factors including *Ins2*, *Mafa*, *Pdx1*, *Nkx2*.*2*, *Nkx6*.*1* and *Glut2* (n = 4). Data presented as mean ± SEM, where p<0.05 was considered statistically significant.

Adherent cultures of pancreatic islets have reduced function *in vitro* [6], and thus we wanted to utilize this culture condition to examine the baseline gene expression of islets. To determine whether the gene expression profile of important β-cell genes and transcription factors changes over time in adherent islet cultures, we performed quantitative PCR at several time points using dissociated rat islets (**Fig 1A**), as well as whole rat islets (**S1 Fig**) in adherent culture plates over a time course of 4 weeks in low glucose (5 mM).

We found that there was a significant downregulation of the β-cell transcription factor and gene expression profile over time for genes including *Ins2*, *Nkx2*.*2*, *Nkx6*.*1*, *Ucn3 and Glut2* normalized to *25S* subunit (**Fig 1A and S1 Fig**). Since these results were performed under glucose levels lower than physiological levels for the rat islets and thus might be a stressful condition, we wanted to determine whether reduction of gene expression also occurred at physiological glucose levels of 10 mM glucose. Similarly, the *in vitro* reduction in β-cell gene expression profile was detected when whole rat islets were cultured under physiological glucose levels for rat islets (11 mM), which is higher compared to human islets (**Fig 1B**) [29]. These results suggest that the gene expression profile of β-cell transcription factors is reduced over time in adherent cultures of pancreatic rat islets.

### Pancreatic extracellular matrix enhances β-cell gene expression *in vitro*

To identify the potential effect of pancreatic decellularized matrix (DCM) proteins on islets, we initially perfused the pancreata of cows at the time of sacrifice in the slaughterhouse to obtain large quantities of bovine pancreas DCM. Bovine DCM was cultured with rat pancreatic islets for 3 weeks to determine whether bovine pancreas DCM can enhance β-cell gene expression. These data showed that bovine DCM enhanced the expression of β-cell genes in dispersed rat islet monolayers; however, this effect was modest when compared to rat pancreatic DCM (data not shown). These initial studies indicated that there could be species-specific effects, including bovine proteins that could be toxic or inhibitory to rat islets. Thus, we abandoned the bovine pancreas approach and instead generated rat pancreatic DCM by perfusion and decellularization of ~100 adult rat pancreata. We adapted a previous protocol for pancreatic decellularization while preserving protein-protein interactions [25]. Cell culture plates were coated with whole pancreatic rat DCM for 2 hours at 37 degrees, and then rat pancreatic islets were cultured on the coated plates for 24 days. A significant increase in the expression of key β-cell genes and transcription factors relative to *25S* subunit was detected in islets cultured with rat pancreatic DCM compared to controls by the end of the culture period, suggesting the maintenance of a stable β-cell gene expression (**Fig 1C**). These results demonstrated that pancreatic rat DCM can maintain the β-cell gene expression profile of rat islets, suggesting that the pancreatic *in vivo* microenvironment contains soluble factors required for maintenance of a stable β-cell gene expression profile.

### Fractioned DCM can recapitulate whole DCM effect on β-cell gene expression

**Fig 2:**
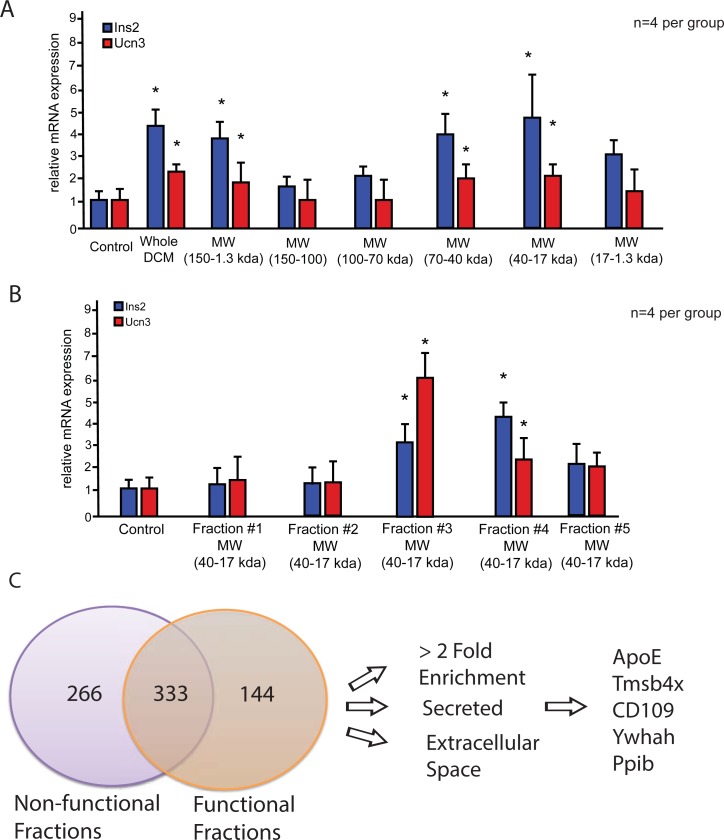
DCM gel filtration fractions recapitulate whole DCM effect on β-cell gene expression. **A)** qPCR analysis of dissociated islet cells cultured for 14 days under low glucose (5 mM) conditions with DCM fractions obtained from gel filtration fractionation of whole DCM. Fraction combinations with M.W 70–40 and 40–17 kDa significantly increase the expression of key β-cell markers Ucn3 and Ins2 and recapitulate the significant increase in expression seen in cells cultured on whole DCM. **B)** Individual concentrated gel filtration fractions with M.W. 40–17 kDa significantly increased the expression of Ucn3 and Ins2. All gene expression levels are relative controls cells. **C)** Venn diagram showing number of proteins based on relative intensity of each protein found in functional fractions as divided by the relative intensity of the proteins found in the non-functional fractions. These proteins were further selected for > 2 fold enrichment, secreted proteins and enrichment in the extracellular space. Data presented as mean ± SEM, where p<0.05 was considered statistically significant, (n = 4).

To determine whether specific soluble factors in the DCM are responsible for the effects on maintaining β-cell gene expression, we fractionated the whole DCM using size exclusion chromatography, to isolate fractions with beneficial effects on cultured β-cells based on molecular weight. Combinations of fractions were used to coat cell culture plates for two hours at 37 degrees. Dispersed islet cells were then cultured on the coated plates for a two- week period and analyzed for the gene expression profiles. *Ucn3*, which has been shown to be a marker for mature β-cells that release insulin upon glucose stimulation, along with *Ins2* expression were used to screen for functional fractions [30]. Fractions containing proteins with molecular weight (MW) between 17 kDa and 40 kDa were found to significantly upregulate both *Ins2* and *Ucn3* expression (**Fig 2A)**. Importantly, these functional fractions recapitulated the upregulation of *Ucn3* and *Ins2* expression that was observed by the whole unfractionated DCM. To further narrow down the effect of DCM to individual functional fractions, we concentrated individual functional fractions, Fraction #1—Fraction #5, and these fractions still induced expression of *Ucn3* and *Ins2* **(Fig 2B)**. Concentrated fractions, Fraction #1 –Fraction #5, corresponding to proteins between 17–40 kDa showed significant increases in *ucn3* and *ins2* expression **(Fig 2B)**.

To attempt identification of candidate proteins within these fractions that could maintain β-cell gene expression, we performed isobaric multiplexed quantitative mass spectrometry of functional fractions along with non-functional fractions to identify enriched proteins within the functional fractions [27]. We identified 144 proteins that were enriched in the functional fractions compared to the non-functional fractions (**Fig 2C**). To narrow down the list of potential candidate proteins, we used several criteria for selection of proteins. First, we selected proteins that were > 2 fold enriched in the functional fractions. Second, we selected proteins that are secreted based on the presence of a signal peptide in the protein or have been reported to be secreted. Finally, we selected proteins that were reported to be enriched in the extracellular space. This narrowed down the list of proteins enriched in the functional fractions to 5 proteins, which included Thymosin beta-4 (Tmsb4x), Tyrosine 3-Monooxygenase/Tryptophan 5-Monooxygenase Activation Protein (Ywhah), Peptidylprolyl Isomerase B (Ppib), Apolipoprotein E (ApoE), and Cluster of Differentiation 109 (CD109) (**Fig 2C**).

### ApoE increases the expression of β-cell gene expression *in vitro*

**Fig 3:**
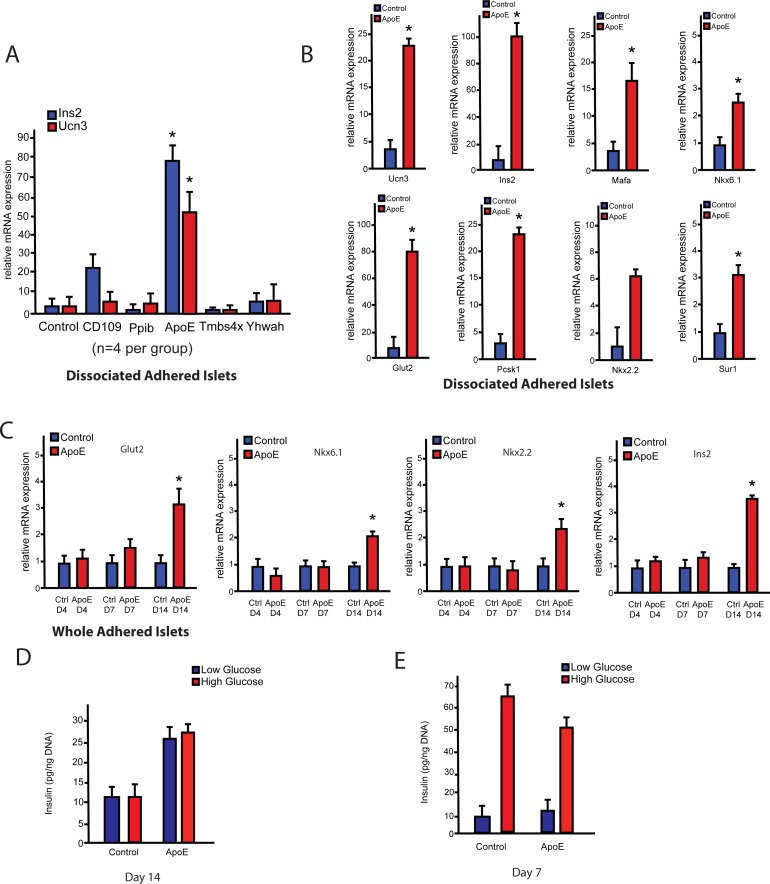
ApoE enhances β-cell gene expression. **A)** Dissociated adhered islets were treated for 14 days under low glucose (5 mM) conditions with recombinant proteins identified through mass spectrometry of functional fractions. Only ApoE significantly upregulated *Ins2* and *Ucn3* expression controls. **B)** Dissociated adhered islets were cultured for a period of 14 days under low glucose (5 mM) conditions and either treated with or without ApoE. ApoE significantly increased the expression of key beta cell markers including *Ucn3*, *Ins2*, *Mafa*, *Nkx6*.*1*, *Pcsk1*, *Sur1* and *Glut2*. Notably, increase in the expression of *Nkx2*.*2* was also observed although not significant. **C)** Whole adhered pancreatic islets were cultured for a period of 14 days under low glucose (5 mM) conditions with or without ApoE. ApoE treatment did not significantly increase β-cell gene expression relative to controls until D14. *Glut2*, *Nk6*.*1*, *Nkx2*.*2 and Ins2* did not show significant increases relative to controls until D14 (n = 4). **D)** Whole rat pancreatic islets were cultured for a period of 14 days with and without ApoE. Glucose stimulated insulin secretion (GSIS) was measured by providing a low glucose (2.8 mM) or high glucose (16.8 mM) challenge and measuring insulin secretion by ELISA. Secreted insulin concentrations were normalized to islet DNA content. ApoE-treated islets showed no significant changes in insulin release upon glucose stimulation compared to controls (n = 4). **E)** GSIS data for rat pancreatic islets cultured in large non-sticky flasks in suspension for a period of 7 days under high glucose (11 mM) conditions with and without ApoE. ApoE-treated islets show comparable levels of insulin release as controls by day 7 (n = 6). Data presented as mean ± SEM, where p<0.05 was considered statistically significant.

In order to determine the effect of the candidate proteins on β-cells, dissociated pancreatic islets were plated and treated with each individual protein. RNA was collected after a two-week culture period to measure gene expression of *Ucn3* and *Ins2*. Only human recombinant ApoE (hrApoE) protein caused a significant increase in the expression of both *Ucn3* and *Ins2* (**Fig 3A**). Given the increase of expression of *Ucn3* and *Ins2* in ApoE treated cells over non-treated controls, we wanted to determine whether ApoE affected the gene expression of β-cells. A significant increase in the expressions of *Ucn3*, *Ins2*, *Mafa*, *Nkx6*.*1*, *Glut2*, *Pcsk1 and Sur1* was observed (**Fig 3B**). *Nkx2*.*2* expression tended to be higher, but not statistically significantly (**Fig 3B**). This experiment was repeated using cultured whole islets instead of dissociated islets to determine whether ApoE also affects whole islets **(S2A Fig)**. Similar to the dissociated islet results, significant increases in the expression of β- cell genes were observed in whole islets cultured with ApoE recombinant protein **(S2A Fig)**.

To determine the effect of hrApoE on β-cell gene expression over time, we performed a time course experiment where whole adhered islets were either treated with or without ApoE for 4, 7 and 14 days **(Fig 3C).** Significant increases were observed in ApoE treated whole adhered islets for the expression of *Glut2*, *Nkx6*.*1*, *Nkx2*.*2* and *Ins2* by day 14. Furthermore, we wanted to determine the difference in gene expression levels between the different islet conditions, and to what extent can ApoE maintain the β-cell maturation gene expression profile. Thus, we cultured dissociated islets, whole adhered islets and in suspension for 14 days with and without ApoE, and compared them to fresh islets. There was a significant decrease in the β-cell gene expression profile in all culture conditions compared to fresh islets. Interestingly, ApoE was able to enhance this gene expression profile comparable to fresh islets. These results suggest that ApoE can enhance and maintain the β-cell gene expression profile to comparable levels of fresh islets (**S2B Fig**).

To determine whether this increase in gene expression was accompanied by improved β-cell function, we performed a glucose stimulated insulin secretion (GSIS) assay to determine the ability of adherent β-cells to release insulin upon glucose stimulation. Similarly, control and ApoE-treated islets were cultured for 14 days, and then the GSIS assay was performed. Control islets showed an inability to respond properly upon glucose stimulation. In addition, ApoE-treated islets showed comparable levels of insulin release to controls upon low and high glucose stimulation after 14 days in adherent culture compared to controls, indicating that ApoE did not change the GSIS response between low and high glucose stimulation (**Fig 3D**). These results suggest that ApoE can only maintain β-cell gene expression and prevent dedifferentiation that takes place in adherent cultures, but with no significant changes on insulin release.

Furthermore, we wanted to examine the effect of ApoE on islets suspended in culture, since islets suspended in culture have been reported to maintain function in culture over time [18]. Control and ApoE-treated islets were suspended in culture for 7 days, and then the GSIS assay was performed. By 7 days, the ApoE-treated islets showed comparable levels of insulin release upon stimulation with low glucose and high glucose (**Fig 3E**). No significant changes in β-cell gene expression were detected for control and ApoE-treated islets in suspension as well (**S3A Fig**). These results suggest that ApoE has no significant effect when islets are cultured under these improved conditions, but can maintain the mature β-cell gene expression profile under dedifferentiation and stressful conditions.

In order to determine whether ApoE is also able to exhibit this effect on human islets under de-differentation conditions, we plated human islets onto 804-G coated 24 well plates for 7 days with or without ApoE supplemented into the medium. While human islets behaved variably in culture, we did observe a trend toward higher Insulin and MafA expression in 2 of 3 experiments, although this was not statistically significant (**S3B Fig**). We speculate that this variability is due in part to expansion of the mesenchyme during this treatment, which decreased the proportion of surviving β- cells over the 7-day treatment period. Interestingly, we observed less Ucn3 expression in the human islets during ApoE treatment. This could possibly be due to the altered pattern of Ucn3 expression in human islets, which is not restricted to β- cells [31].

### The effect of ApoE on β-cell gene expression is mediated through heparan sulphate proteoglycans, but not the LDL receptor

**Fig 4:**
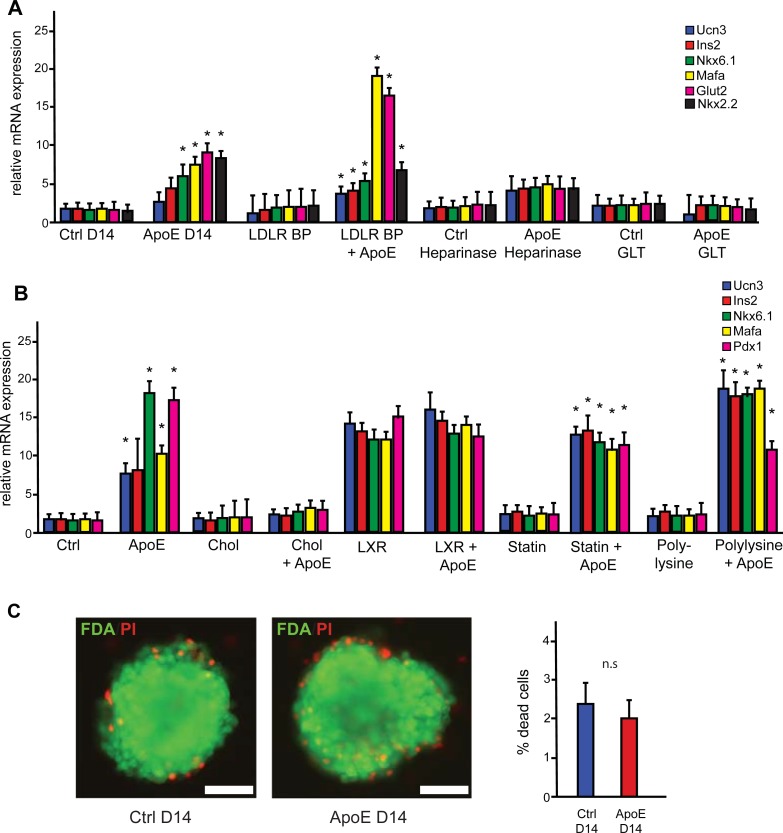
Mechanism of ApoE-mediated effect on β-cells. **A)** Gene expression analysis of pancreatic whole islets in adhered culture for 14 days with ApoE as well as an LDL receptor blocking peptide, Heparinase and high glucose (16.7 mM) with 500 uM (GLT). ApoE significantly increased the expression of key beta cell markers when cultured with the LDL receptor blocking peptide group. In contrast, the heparinase treated group showed no significant changes in gene expression between control and ApoE treated islets. Similarly, GLT conditions also abolished the effect of ApoE on maintaining the β-cell gene expression profile (n = 3–4), in contrast to the ApoE D14 group. **B)** Gene expression analysis of pancreatic whole islets in adhered culture for 14 days with ApoE as well as cyclodextrin loaded with cholesterol, 22R-Hydroxycholesterol, Simvastatin and Polylysine. High levels of cholesterol abolished the effect of ApoE on maintaining the β-cell gene expression profile, while LXR activation by 22R-Hydroxycholesterol enhanced β-cell gene expression similar to ApoE. Neither Simvastatin nor Polylysine alone had a significant effect on β-cell gene expression, (n = 3–4). **C)** Viability assay of islets showing no significant changes between the number of viable and dead cells in both control and ApoE-treated islets (n = 10). Scale bar, 50 uM. Data presented as mean ± SEM, where p<0.05 was considered statistically significant and n.s. means not significant.

To further dissect the mechanism by which ApoE exerts its effect on β-cells, we wanted to determine whether this effect is mediated through the LDL receptor. Thus, we cultured whole pancreatic islets in adherence with and without ApoE together with an LDL receptor blocking peptide. Blocking the LDL receptor had no effect on the increase in gene expression profile with ApoE, possibly due to the expression of other members of the LDL receptor family (**Fig 4A**).

ApoE has been demonstrated to bind to heparan sulphate proteoglycans on the cell surface and extracellular matrix to exhibit its function [32]. To further elucidate whether ApoE is dependent on heparan sulphate proteoglycans (HSPGs) to maintain the β-cell gene expression profile, we cultured whole adhered pancreatic islets with heparinase, which disrupts HSPGs. Interestingly, treatment of islets with heparinase abolished the ApoE-mediated increase in β-cell gene expression, suggesting that the effect of ApoE is partly mediated through HSPGs (**Fig 4A**).

Owing to the lipid-clearing role of ApoE, we wanted to determine whether ApoE can ameliorate the effect of glucolipotoxicity on pancreatic islets. Thus, we cultured whole adhered rat islets under high glucose 16.7 mM and palmitate 500 uM (GLT) with and without ApoE. We found that ApoE was insufficient to maintain the β-cell gene expression profile and reduce the toxicity of GLT conditions, in contrast to the ApoE D14 group (**Fig 4A**).

To further dissect whether the effect of ApoE on β-cell gene expression is related to its role as a lipid transporter, we cultured adhered whole pancreatic islets with cyclodextrin loaded with cholesterol (50 mg of Cholesterol in 1 g of methyl β-cyclodextrin). Interestingly, increased cholesterol levels abolished the effect of ApoE on β-cell gene expression suggesting that increased cholesterol levels might impact the effect of ApoE on β-cell gene expression (**Fig 4B**). In addition, we treated adhered whole islets with 10 uM 22R-Hydroxycholesterol to stimulate liver x receptor activation. This led to increased gene expression profile similar to ApoE (**Fig 4B**), which is consistent with previous results that LXR stimulation enhances islet function [33]. To determine whether lowering intracellular cholesterol levels and stimulating SREBP gene expression affects β-cell phenotype, we treated the islets with 1uM Simvastatin. There was no effect on β-cell gene expression, in contrast to ApoE treated islets (**Fig 4B**). This corroborates previous findings, which showed that simvastatin reduced insulin secretion in islet cultures [34]. Similarly, polylysine treatment of islets to mimic the binding of ApoE to HSPGs had did not enhance their gene expression profile, suggesting that ApoE signaling is required together with HSPGs (**Fig 4B**). Collectively, these results reveal a dynamic role for ApoE in regulating β-cell gene expression via dependent and independent roles as a lipid carrier.

To determine the overall impact of ApoE on islet viability, we performed viability staining using fluorescein diacetate (FDA) and propidium iodide (PI) to quantify whole adhered islet cell viability. No differences were detected in the number of dead cells between controls and ApoE-treated islets, suggesting that the effect of ApoE on pancreatic islets is not due to enhancement of islet cell viability (**Fig 4C**). In addition, BrdU treatment showed that there was minimal DNA synthesis and proliferation in both control and ApoE-treated whole adhered islets (**S4 FIg**). These results suggest that ApoE does not affect islet cells through cell viability and proliferation.

### The ApoE-beta cell effect is dependent on JAK/STAT signaling

**Fig 5:**
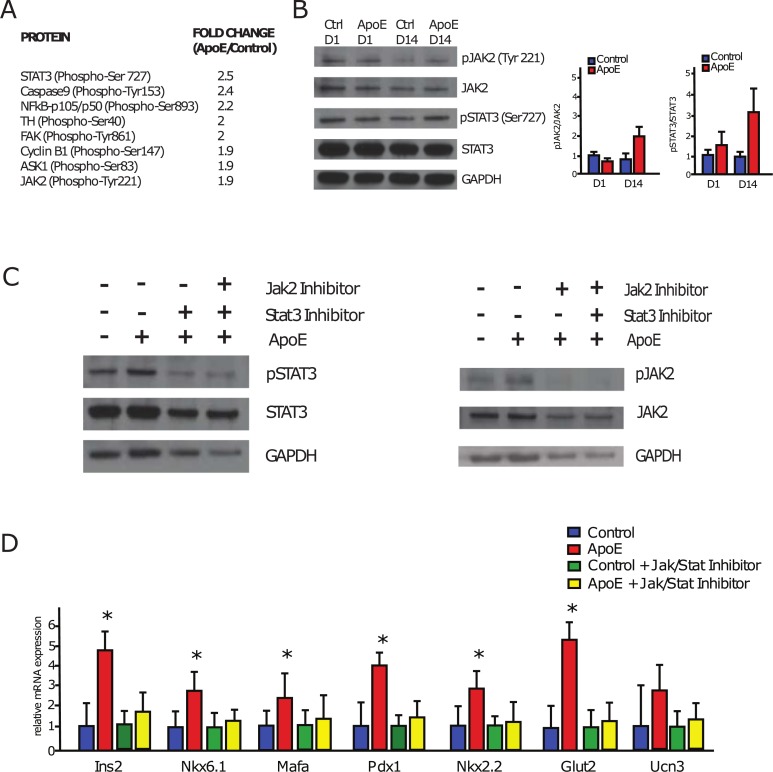
Effect of ApoE on β-cells is dependent on JAK/STAT signaling. **A)** List of upregulated proteins from the phospho explorer array from ApoE-treated whole islets in adherence compared to controls. Fold changes are considered significant when they are greater than 1.5. The results of the Antibody Array Assay identified STAT3 (phosphorylated at Ser727) as the most-upregulated protein. JAK2 (phosphorylated at Tyr221) was also among the most upregulated proteins. **B)** Left, Western blot analysis of phosphorylated STAT3 and JAK2, confirming the array results showing a nearly two-fold increase in the ApoE treated islets at day 14. Right, quantification of bands using Image J software. **C)** Western blot analysis using STAT3 and JAK2 inhibitors, which demonstrates that these inhibitors can inhibit the phosphorylation of these proteins in whole adhered islets at day 14 with or without ApoE. **D)** Gene expression analysis of whole adhered pancreatic islets cultured for 14 days with ApoE as well as STAT3 and Jak2 inhibitors. As expected, ApoE treatment significantly increased β-cell gene expression relative to controls, while this increase was abolished in islets treated with STAT3 and JAK2 inhibitors. Data presented as mean ± SEM, where p<0.05 was considered statistically significant (n = 4).

ApoE is a 34 kDa protein that has been shown to play essential roles in lipid transport, lipid metabolism, neuronal function, immune system regulation, and various roles as an important signaling molecule [35]. Although much is known about the role of ApoE in the liver and brain, less is known about its role on pancreatic islets and downstream signaling pathways. Interestingly, pancreatic islets from ApoE knockout mice show reduced function upon glucose stimulation [36]. These results suggest that ApoE might be required for proper islet function, but the mechanism remains unclear. To determine the mechanism by which ApoE maintains β-cell gene expression, we performed a phospho protein assay on control and ApoE-treated whole rat islets in adherence after 14 days to identify potential signaling pathways regulated by ApoE. The results showed that Stat3, phosphorylated at Serine 727, was the most upregulated phosphorylated protein in the ApoE-treated islets (**Fig 5A**). Furthermore, Jak2, phosphorylated at Tyrosine 221, was upregulated as well (**Fig 5A**). This suggests the potential activation of the Jak2/Stat3 pathway in ApoE-treated cells. These results were confirmed by western analysis where quantification of western blots show a nearly two-fold upregulation of phosphorylated STAT3 and Jak2 in islets treated with ApoE for 14 days compared to controls from two independent experiments, which are comparable to the levels of phosphorylated STAT3 and JAK2 of day 1 islets suggesting that ApoE was sufficient to maintain STAT3 and JAK2 phosphorylation in culture (**Fig 5B**).

To determine whether the effect of ApoE on β-cells is dependent on the JAK/STAT signaling pathway, we used inhibitors of STAT3 (Pp-YLKTK-mts) and JAK2 (AG490) to block phosphorylation and activation of these proteins. The inhibitors were able to block the phosphorylation of STAT3 and JAK2 as confirmed by western analysis (**Fig 5C**). To identify the overall impact of JAK/STAT inhibition on islet cell viability, we performed a viability assay in whole islets in adherence treated with JAK/STAT inhibitors. The inhibitors did not affect islet viability in both control and ApoE treated islets (**S5 Fig**). To determine if inhibition of STAT3 and JAK2 phosphorylation abolished the effect of ApoE, we treated whole adhered islets with ApoE and inhibitors for 14 days *in vitro*. As expected, islets treated with ApoE showed an upregulation in the β-cell gene expression of *Ucn3*, *Ins2*, *Nkx6*.*1*, *Mafa*, *Pdx1*, *Nkx2*.*2* and *Glut2* (**Fig 5D**). In contrast, the islets treated with both ApoE and the inhibitors of STAT3 and JAK2 showed no significant changes in the expression of these genes (**Fig 5D**), revealing that ApoE enhances β-cell gene expression profile at least partly through phosphorylation of STAT3 and JAK2.

## Discussion

Our study shows that pancreatic islets in adherent cultures exhibit downregulation of key β-cell genes and transcription factors necessary for the maintenance of the mature β-cell gene expression. This observation is consistent with the reduced function of islets in adherent cultures, as well as the lower success rates in diabetic patients and mice that receive cultured islets versus fresh islets [37, 38]. These results suggest that important signals from the microenvironment are required for maintenance of β-cell function [6]. Extracellular matrix components have been shown previously to enhance both survival and function of pancreatic islets; however, the various extracellular matrix factors used were not designed to mimic the native pancreatic microenvironment [9–12]. Furthermore, the use of organ matrices as a way to improve cellular differentiation and maturation in the construction of tissues has been successful [39, 40]. In this study, we demonstrate that whole pancreatic DCM increased expression of key β-cell genes and transcription factors of β-cells and blocked dedifferentiation of islets in adherent cultures.

Following protein fractionation and mass spectrometry of pancreatic extracellular proteins we identified ApoE as an extracellular factor that can recapitulate the effect of DCM on β-cell gene expression. Interestingly, the gene expression pattern in GEO profiles of diabetes models in both mouse and human reveal a differential expression of ApoE levels during diabetes, and reveal an important connection between ApoE and diabetes [41]. In addition, islets from ApoE knockout mice have reduced function and have a significantly lower GSIS response [36]. Since lack of ApoE results in increased levels of cholesterol and lipids, the reduced β-cell function has been attributed to lipotoxicity in the ApoE knockout mice, since high levels of lipids and cholesterol have been shown to affect β-cell function [42]. Thus, it remains difficult to distinguish between the lipid dependent and lipid independent roles of ApoE in diabetes. Furthermore, when cholesterol is added together with ApoE, this abolished the effect of ApoE on β-cell gene expression. Interestingly, LXR stimulation with Oxysterol had a similar effect to ApoE on β-cell gene expression. Thus, our study demonstrates that the lipid-free ApoE recombinant protein is capable of enhancing β-cell maturation, suggesting that ApoE might enhance β-cell gene expression and stability through both independent and dependent roles as a lipid carrier. These experiments utilized rat islets, and we have not yet studied relative changes within each islet that may be occurring. Experiments are underway to determine the effects of ApoE on individual cell types within the islet. In addition, we have shown that the effect of ApoE on maintaining β-cell gene expression is mediated through binding to HSPGs, suggesting that this interaction is required for exerting its effect on pancreatic islets. It remains to be determined whether ApoE exerts its effect indirectly by binding another factor that enhances β- cell gene expression transcription. Future experiments will aim to decipher potential binding partners for ApoE than can stimulate pancreatic islet maturation and inhibition of dedifferentiation. The increase in β-cell gene expression was not accompanied by an increase in islet function. This suggests that ApoE is insufficient to drive complete functional enhancement of β-cells; however ApoE stimulates an important initial step by increasing the expression of the β-cell transcriptional machinery.

To investigate the downstream signaling pathways by which ApoE acts on β-cells, we performed a signaling pathway analysis, which identified an increased phosphorylation of STAT3 and JAK2. Interestingly, a recent report demonstrated that upregulation of JAK/STAT signaling pathway was associated with ApoE in gastric cancer [43]. More importantly, inhibition of phosphorylated STAT3 and JAK2 abolished the effect of ApoE on β-cells, suggesting that ApoE at least partly improves β-cell gene expression through the JAK/STAT pathway. These results are in line with emerging studies suggesting that liver-derived secreted factors, known as hepatokines, regulate metabolism of different metabolic tissues [44, 45]. Thus, we uncovered a novel role for ApoE as a pancreatic extracellular factor in that it can maintain the mature rat β-cell gene expression, although we did not find that ApoE added to cultured islets could enhance insulin secretion. Whether additional DCM factors are required to maintain β-cell function and maturation is yet to be determined. Nonetheless, the overall maintenance of β-cell differentiation could improve islet transplantation outcomes in diabetes.

In this study, we describe an approach that has the potential to identify soluble extracellular factors with specific functions from the extracellular microenvironment. Elucidating the microenvironment signals that maintain advanced cell functions has implications for improving *in vitro* disease models. Furthermore, understanding factors that maintain cell phenotype may enable improved transplantation strategies.

## Supporting information

S1 Fig Whole islet gene expression profile is lost in adherent cultures in vitro. Gene expression analysis of β-cell transcription factors from rat pancreatic whole islets cultured under low glucose (5 mM) conditions for 2 weeks shows reduced gene expression over time (n = 4).
(EPS)

S2 Fig ApoE enhances islet gene expression profile in vitro. A) Whole rat pancreatic islets were cultured for a period of 14 days with or without ApoE under low glucose (5 mM) conditions. ApoE significantly increased the expression of key β-cell markers including Ins2, Ucn3, Glut2, Nkx2.2, Nkx6.1, Pcsk1, Sur1 and Pc. B) Whole rat pancreatic Islets were cultured either in adhered or suspension cultures, as well as dissociated adhered islets were cultured for 14 days and compared to fresh islets. A significant reduction in β-cell gene expression profile was detected in all culture conditions compared to fresh islets. ApoE led to an enhanced expression of the β-cell genes in the different culture conditions.
(EPS)

S3 Fig Effect of ApoE on islets cultured in suspension and on human islets. A) Whole human pancreatic islets were cultured for a period of 7 days under physiological glucose (11 mM) conditions with or without ApoE. Comparable levels of key β-cell markers was observed.
B) Human islets were cultured for 7 days in 804G coated plates with and without ApoE. Each data point is a qPCR replicate shown with geometric mean, which shows a trend toward higher expression of both Insulin and MafA.
(EPS)

S4 Fig ApoE Treatment does not stimulate islet cell proliferation. Whole rat pancreatic islets were cultured for a period of 14 days with or without ApoE together with BrdU. Very low levels of BrdU positive cells were detected in both groups. Scale bar, 50 uM. Data presented as mean ± SEM, where n.s. means not significant (n = 10).
(EPS)

S5 Fig JAK/STAT inhibition does not affect islet viability. Whole pancreatic rat islets were cultured for a period of 14 days with or without ApoE together with JAK/STAT inhibitors. Comparable levels of viable and dead cells were detected between the two groups. Scale bar, 50 uM. Data presented as mean ± SEM, where n.s. means not significant (n = 10).
(EPS)

S1 File Supporting information data. This file contains the list of primers used in this study and supplementary materials and methods.
(PDF)
