# Supplementary material for: Apolipoprotein E is a pancreatic extracellular factor that maintains mature β-cell gene expression
Source: PLoS One. 2018 Oct 10;13(10):e0204595. doi: 10.1371/journal.pone.0204595 (PMC6179231; doi:10.1371/journal.pone.0204595)
Supplement: S1 File — This file contains the list of primers used in this study and supplementary materials and methods. (PDF) [file pone.0204595.s006.pdf]

Supplemental data

List of primers used for Real time PCR

Gene	Forward Primer	Reverse Primer
**25S**	GTGGTCCACACTACTCTCTGAGTTTC	GACTTTCCGGCATCCTTCTTC
**Ins2**	TCTTCTACACACCCATGTCCC	GGTGCAGCACTGATCCAC
**Ucn3**	ACAGATACCAATCCCAAGCACA	GCAAATTCTTGGCCTTGTCAAT
**Glut2**	TGGGTTCCTTCCAGTTCG	AGGCGTCTGGTGTCGTATG
**Mafa**	CTTCAGCAAGGAGGAGGTCATC	GCGTAGCCGCGGTTCTT
**Nkx2.2**	ATCTGGTTCCAAAACCATCG	TGTACTGGGCGTTGTATTGC
**Nkx6.1**	TCTTGGCTCTCAGGTCTGGT	GGGCTTGTTGTAATCGTCGT
**Pcsk1**	CAGGGGAGACAAGGAAAA	AGGCACTGCTGATAGAGATGG
**Pdx1**	GACACATCAAAATCTGGTTCCAAA	TCCCGCTACTACGTTTCTTATCTTC
**Pc**	TTGAAGGATGTGAAGGGCC	ACCTTTCGGATAGTGCCCTC
**Sur1**	GCAGCCGAGAGCGAGGAAGATGA	ACAGCCAGGGCGGAGACACAGAGTA

Decellularization of Rat Pancreata

SD rats were euthanized, and the abdominal cavity was opened, and the pancreatic duct was immediately clamped. The pancreas was perfused through the hepatic duct with 1x sterile PBS and forceps were used to remove the pancreas from the abdomen. The pancreas was immediately placed in a 1% Triton-X100 and 0.1% NH4OH solution for decellularization of the pancreatic tissue. The decellularization solution was changed every three days for a period of one week. After the decellularization of pancreatic tissue, the decellularized matrix was rinsed with 1x PBS and stored at -20°C.

For trypsin solubilization of lyophilized DCM, 15 mg of trypsin (Gibco Life Technologies Lot #: 1140299) were dissolved in a 30 mL 100 mM solution of ammonium bicarbonate of pH 8. 300 mg of lyophilized DCM was added to the trypsin solution and stirred for 24 hours at 37°C. The digestion reaction was stopped with the addition of 15 mg of trypsin inhibitor (Gibco Life Technologies Lot # 1549820). The solution was centrifuged at 25,000g for 15 minutes at 4°C, and supernatant was filtered using a 0.45μm syringe filter. A final concentration of ~4.7 mg/mL was obtained from the trypsin digestion. DCM solution was stored at 4°C for short-term use.

Real-Time PCR

Total RNA was isolated from rat pancreatic islets using Trizol (Invitrogen) and reverse transcription for cDNA synthesis was performed using random hexamer primers with the High-Capacity cDNA Reverse Transcription Kit (Invitrogen). Real time PCR was performed with SYBR Green (Applied Biosystems) on CFX384 Touch Real-Time PCR detection system (Bio-Rad). 25S was used as a loading control to normalize gene expression using the $\Delta\Delta$Ct method.

Gel Filtration Fractionation of Digested DCM

Gel filtration chromatography was performed on solubilized decellularized matrix solutions in order to separate total proteins in digested DCM by protein size. Several fractions were obtained from gel filtration chromatography each containing different size proteins from the DCM. Bio-Rad BioLogic Duo-Flow Chromatography system was used for the gel filtration fractionation of the DCM proteins. A calibrated Superdex 200 column was used for the fractionation process according the manufacturers protocol. A gel filtration protein standard (Biorad) was run through the column for the determination of protein sizes obtained from fractionation. After standard run, the column was washed for one and a half hours with 1x PBS. 5 mL of either pepsin or trypsin solubilized DCM was loaded onto the column followed by automated fractionation of total proteins in these solutions by size. Obtained fractions were stored either at 4°C for short-term use, or at -20°C for long-term storage.

Protein Extraction

Cells were centrifuged at 530 xg for 2 minutes at 4°C. The cells were then washed with ice cold PBS by centrifuging at 530 xg for 5 minutes at 4°C; this was repeated twice. A mixture was made that, for every 1mL, contained: 860 μL dH_2O, 100 μL RIPA Buffer 10X, 10 μL Phosphatase Cocktail 2, 10 μL Phosphatase Cocktail 3, 10 μL PMSF, and 10 μL Protease Inhibitor. 300μL of this mixture was added per sample (for each 10 mm cell-culture plate). The pellet was resuspended in this mixture and incubated on ice for 30 minutes, with vortexing at 10 minute intervals. The mixture was then centrifuged at 14,000 xg for 15 minutes at 4°C. The supernatant was collected and stored at -80°C.

Western Analysis

Protein concentration was measured using the Thermo Scientific Pierce™ BCA Protein Assay Kit to ensure that equal amounts of protein are used for each sample. Samples were loaded to the wells of sodium dodecyl sulfate polyacrylamide (SDS-PAGE). A polyvinylidene diflouride (PVDF) membrane was preactivated in methanol for 1 minute and then rinsed in Transfer Buffer (1X) and used for transfer in the BioRad Semidry Transfer Box. The transfer was conducted at 25V for 35 minutes. After the transfer, the PVDF membrane was blocked in 5% Bovine Serum Albumin (BSA) TBST (Tris-Buffered Saline-1% Tween 20) for 1 hour. The PVDF membrane was incubated in specific dilutions of primary antibodies (GAPDH, Cell Signaling, pStat3-S727, Cell Signaling, pJak2-Y221, Cell Signaling, STAT3, Transduction Laboratories, JAK2, Cell Signaling) overnight at 4°C. The membrane was washed 3 times in TBST (1X) for 5 minutes then incubated in secondary antibody for 1 hour on a rotating platform at room temperature. The membrane was visualized using Western Lightning *Plus*-ECL (Enhanced Chemiluminescence Substrate). Quantification was performed using Image J software.

Glucose Stimulated Insulin Secretion (GSIS)

For islets cultured in suspension, 10 islets were handpicked for each biological replicate. Islets were washed twice in Krebs Ringer Bicarbonate Hepes Buffer (Krb buffer). Next, islets were incubated for 1 hr in low (2.8 mM) glucose Krb buffer at 37°C. Krb was removed and discarded and islets were then incubated again in low glucose Krb buffer for 1 hr at 37°C. After this incubation supernatant was collected for low glucose insulin secretion measurement. Low glucose buffer replaced by high glucose Krb buffer (16.8 mM) for measurement of insulin secretion at high glucose. Secreted insulin

concentrations were measured using Alpco's Ultrasensitive Rat Insulin ELISA kit following the manufacturer's protocol. The ELISA plates were read on a SpectraMax M5 machine, for the determination of secreted insulin concentrations. After glucose challenges, islets were collected for measurement of DNA content for the normalization of secreted insulin concentrations to total islet DNA content. DNA was analyzed using the Cyquant Cell Proliferation Kit (Life Technologies) according to the manufacturer's protocol. Measurement of DNA concentration was done by fluorescent reading by the SpectraMax M5 machine. Secreted insulin concentrations were normalized to DNA concentrations.

BrdU Treatment and Immunocytochemistry

Rat pancreatic islets were treated with BrdU (10 uM) labeling solution for 14 days in culture with and without ApoE. Islets were then washed with PBS, digested with 0.1% trypsin, and fixed with 70% ethanol for 30 minutes. Cells were then added to Poly-L-Lysine coated chamber slides. DNA was denatures by adding 2N HCl for 1 hour at room temperature. Cells were washed with PBS, blocked with 10% goat serum for 2 hours at room temperature. Slides were incubated with primary antibodies against BrdU (mouse monoclonal, abcam) and Insulin (rat polyclonal, Hybridoma bank) at 4 degrees overnight. Slides were washed with PBS 3 times, and incubated with a goat anti-mouse secondary antibody conjugated to Alexa Fluor 488 and a goat anti-rat secondary antibody conjugated to Alexa Fluor 555 for 1 hour in dark at room temperature. Slides were then washed with PBS 3 times; DAPI was added then slides were mounted.

Human Islet Culture

Human islets were plated on 804G-coated 24-well plates in triplicate. Cells were maintained in CMRLS medium with or without 2 μg/mL recombinant human ApoE.

Medium was changed every 48 hours. After 7-10 days of culture, RNA was collected in RLT and extracted using RNEasy RNA extraction columns. cDNA was generated using Superscript III cDNA reaction kit. Real Time RT-PCR was performed using Taqman Probes designed against Insulin, Ubiquitin C, MafA, and Ucn3 and run in an ABI7900HT instrument. Fold changes were calculated using delta Ct.

Statistical Analysis

Results are expressed as mean ± SEM. An unpaired Student's *t* test was used to determine statistical significance of all samples. *$P<0.05$, **$P<0.01$ were considered statistically significant.
